# Spontaneous hyphema associated with ocular syphilis

**DOI:** 10.1186/s12348-020-00209-z

**Published:** 2020-07-27

**Authors:** Brent Deibert, Kellie Wark, Rocio Diaz, Christopher Blodi

**Affiliations:** 1 University of Nebraska Medical Center, Truhlsen Eye Institute, 3902 Leavenworth St, Omaha, NE 68105 USA; 2 Department of Infectious Diseases, Kansas University Medical Center, Kansas City, USA

**Keywords:** HIV, Hyphema, Neurosyphilis, Ocular syphilis, Syphilis, Uveitis

## Abstract

The purpose of this case series is to illustrate a novel presentation of ocular syphilis. Two cases of ocular syphilis presenting with spontaneous hyphema are described, demonstrating that spontaneous hyphema can be caused by ocular syphilis. This association has not previously been reported.

## Introduction

Spontaneous hyphema has been reported in cases of anterior uveitis [1]. However, review of literature found no reported cases of spontaneous hyphema associated with ocular syphilis. Here we describe two such cases.

## Case reports

### Case 1

A 32-year-old man presented with 2 weeks of painful and blurry vision in his right eye (OD) with watery discharge. His social history was notable for sex with men, with one sexual partner in the past year. He reported safe sexual practices. His urine tested positive for amphetamines. He denied use of illicit intravenous drugs. Vision was 20/400 OD and 20/70 in the left eye (OS), improving to 20/200 and 20/30 with pinhole. The intraocular pressure (IOP) was normal in both eyes (OU) and there were no relative afferent pupillary defects (RAPD) OU. He had new heterochromia, with a dark green iris OD and a light blue iris OS. No neovascularization of the iris was seen. Slit lamp exam OD revealed upper and lower eyelid edema, moderate injection of the conjunctiva, corneal edema with keratic precipitates and a disorganized hyphema attached to the peripheral iris. There was a significant degree of anterior chamber cell and flare, along with dense vitritis and optic nerve edema. The left eye showed signs of mild anterior uveitis only. The patient was diagnosed with panuveitis OD and anterior uveitis OS.

Blood tests revealed a positive RPR (1:256), positive Treponema pallidum antibody, HIV-1 antibody with CD4 count of 36, and CMV IgG. Blood tests were negative for CMV IgM, Tuberculosis interferon antigen, Toxoplasma gondii IgM and IgG, and lysozyme. Other abnormal findings were leukopenia, an elevated ESR (> 128 mm/hr), and elevated liver enzymes.

The patient was admitted and started on a darunavir, cobicistat, emtricitabine, tenofovir and alafenamide combination tablet based on his HIV resistance profile. Syphilis was treated with intravenous (IV) Benzylpenicillin 4 million units every 4 h for 14 days followed by three weekly benzathine penicillin 2.4 million units intramuscular (IM) injections, given his degree of immunosuppression.

Ten days later, his vision OD was 20/300 with pinhole improvement to 20/125. There was no RAPD and IOP was normal. The conjunctiva was clear and there was no sign of anterior uveitis OU and no residual hyphema. Iris heterochromia had resolved. Moderate posterior uveitis persisted OD with a temporal vitreous snowball. The disc was slightly hyperemic and the periphery of the retina had salt and pepper markings, consistent with RPE hypertrophy and hypotrophy. An OCT showed an epimacular membrane with vitreo-macular traction and foveal distortion. Topical prednisolone 1% four times a day ODand topical atropine 1% twice a day OD were started.

OD had 20/400 visual acuity with pinhole improvement to 20/125 2 weeks later. Vitreous cell activity was reduced with partial resolution of vitreous snowball. He was then lost to follow-up.

### Case 2

A 47-year-old man complained of poor balance for 1 month and was found to have a low sodium of 129 mmol/L. He was admitted elsewhere, and sodium was corrected with fluid restriction but this did not improve his imbalance. A neurological consultation suspected that his imbalance was secondary to neuropathy and started him on appropriate therapy. He was discharged shortly after. His inflammatory markers were elevated but this was not followed up.

Two days after discharge, he developed painful vision loss OD and diffuse joint and back pain. There was no history of prior eye surgery or trauma. Exam OD was notable for light perception vision without RAPD, neovascularization of the iris, and 1 mm hyphema and inflammation in the anterior chamber. Due to dense inflammation, there was no view of the fundus OD. B-scan revealed choroidal thickening and vitreous opacities representative of inflammation or hemorrhage OD. OS vision was 20/70 without pinhole improvement. There was no RAPD OS and slit lamp exam showed keratic precipitates and anterior inflammation. There was optic nerve edema OS. IOP OD was 10 mmHg and 11 mmHg OS. He was prescribed topical prednisolone drops 1% every 2 h OU and topical cyclopentolate 1% two times per day OU.

Blood tests were positive for HIV, RPR (1:128), and Treponemal antibody. Tuberculosis interferon, Angiotensin converting enzyme, ANA blood tests were negative. Given his constellation of symptoms neurosyphilis was suspected. Social history revealed that he was sexually active with multiple female partners in the past month and did not use condoms. He also had a history of past IV drug use and was currently smoking methamphetamine.

Lumbar puncture was also positive for Treponemal antibody. IV Benzylpenicillin 4 million units every 4 h was administered for 14 days on an inpatient basis. HIV screening during this admission was positive and he was started on highly active antiretroviral therapy.

Follow-up 3 weeks later was notable for hyphema resolution but persistent keratic precipitates. OD vision was greatly improved to 20/200. OS was 20/40 with dense posterior synechiae, cataract, and a fixed mid-position pupil. IOP 10 mmHg OD and 9 mmHg OS. There was no sign of active inflammation OU. Prednisolone and cyclopentolate were stopped. It was thought that he had a visually significant nuclear and cortical cataract, posterior synechiae, and pupillary membrane OD. Cataract surgery was performed OD. A month after cataract surgery, OD vision was 20/100 without pinhole improvement and exam revealed trace cell in the anterior chamber, vitreous pigment, and a mild epiretinal membrane with subtle edema on OCT. He was re-started on prednisolone 1% four times a day OD and referred to Retina. At 6 months from initial presentation, he was seen by Infectious Disease and HIV RNA was undetectable and his RPR had declined enough to be considered clinically cured of neurosyphilis. The patient was lost to Ophthalmology follow up and all efforts to contact him have failed.

## Discussion

Spontaneous hyphema has been described as a rare symptom of anterior uveitis. Duke-Elder and Perkins described 3 mechanisms in which hyphema can result from uveitis: leakage from neovascularized iris vessels, vessel damage secondary to vasculitis, or increased diapedesis of inflammatory cells [2]. Fong and colleagues reported 5 cases of spontaneous hyphema associated with uveitis, but none of these cases had syphilis as the underlying cause [1]. Only 3 of the 5 cases showed iris neovascularization [1].

Uveitis most commonly occurs in the secondary and tertiary stages of syphilis [3]. The most common manifestations of ocular syphilis are granulomatous iridocyclitis (46% syphilitic uveitis cases), non-granulomatous iridocyclitis (25%), panuveitis (13%), posterior uveitis (8%) and keratouveitis (8%) [3–8]. Although syphilis was one of the most common causes of uveitis in the first half of the twentieth century, rates dropped dramatically with the advent of penicillin treatment [3]. Retrospective review of uveitis etiologies at a New York City referral center found that 8% of uveitis cases (44 of 552) were attributed to syphilis and two international multicenter retrospective reviews demonstrated similar rates, although another United States referral center attributed only 2.45% (25 of 1020) of uveitis cases to syphilis [4–6, 9].

The literature is divided in terms of whether the ocular findings at presentation or the visual outcomes in syphilis differ when there is HIV co-infection [3, 6, 10–12]. Several case reviews have demonstrated higher frequency of placoid chorioretinitis (30%), optic nerve involvement (25%), and necrotizing retinitis (35%) when HIV co-infection is present [13, 14]. Additionally, Tran and colleagues reported worse visual outcomes and higher rates of relapse in the HIV population despite standard treatment [13].

## Conclusions

Ocular syphilis can present with a wide variety of non-specific and specific signs and symptoms. These two cases add to this list of presenting signs demonstrating that spontaneous hyphema can occur with ocular syphilis. These cases also demonstrate that the formation of hyphema associated with syphilis can occur with or without the occurrence of iris neovascularization. Therefore, the presence of hyphema should expand the differential diagnosis to include ocular syphilis.

### Patient consent

Consent to publish the case report was not obtained. This report does not contain any personal information that could lead to the identification of the patient.

## Data Availability

Not applicable, no data was analyzed, all cited references can be found on PubMed.

## Abbreviations

ANA: Antinuclear antibodies
B-Scan: Brightness scan ultrasound
CMV: Cytomegalovirus
ESR: Erythrocyte sedimentation rate
HIV: Human immunodeficiency virus
IgG: Immunoglobulin type G
IgM: Immunoglobulin type M
IOP: Intraocular pressure
IV: Intravenous
mm/hr: Millimeter per hour
mm: Millimeter
mmol/L: Millimoles per liter
OCT: Ocular coherence tomography
OD: Right eye
OS: Left eye
OU: Both eyes
RAPD: Relevant afferent pupillary defect
RNA: Ribonucleic acid
RPR: Rapid plasma regain
